# Cecal Microbial Diversity and Metabolome Reveal a Reduction in Growth Due to Oxidative Stress Caused by a Low-Energy Diet in Donkeys

**DOI:** 10.3390/antiox13111377

**Published:** 2024-11-11

**Authors:** Li Li, Xiaoyu Guo, Yanli Zhao, Yongmei Guo, Binlin Shi, Yan Zhou, Yongwei Zhang, Sumei Yan

**Affiliations:** 1 Inner Mongolia Key Laboratory of Animal Nutrition and Feed Science, College of Animal Science, Inner Mongolia Agricultural University, Hohhot 010018, China; lily972021@emails.imau.edu.cn (L.L.); gxy2024@imau.edu.cn (X.G.); ylzhao2017@imau.edu.cn (Y.Z.); ymguo2020@imau.edu.cn (Y.G.); shibl@imau.edu.cn (B.S.); alicezqy@emails.imau.edu.cn (Y.Z.); 2 Inner Mongolia Grassland Yulv Science and Technology Animal Husbandry Co., Ltd., Hohhot 011500, China; mailzc@emails.imau.edu.cn

**Keywords:** meat donkey, dietary energy, growth performance, cecal microbiome and metabolome, oxidative stress

## Abstract

Dietary energy level plays an important role in animal growth and development. The present study investigated the effect of dietary energy on the growth performance, antioxidative state, and nutrient digestion of meat donkeys. It simultaneously explored the probable reason for cecal microbiota and metabolome. Twelve meat donkeys (male) aged 1 year with a similar weight (150 ± 25 kg) were divided into two treatment groups: low-energy group (E1) and high-energy group (E2). The experiment was divided into a 10-day pre-trial period and a 135-day trial period. Donkeys in the trial periods were fed diets with digestible energy values (in dry matter) of 12.08 and 13.38 MJ/kg (pre-fattening, 1–45 d), 13.01 and 14.27 MJ/kg (mid-fattening, 46–90 d), and 13.54 and 14.93 MJ/kg (late-fattening, 91–135 d). The results show that E1 decreases body weight, average daily gain (ADG), and the digestibility of crude protein, ether extract, neutral detergent fiber, and acid detergent fiber (*p* < 0.05), but increases cecal acetate and butyrate concentrations, non-esterified fatty acids (NEFAs) in serum, and the ratio of dry matter intake to ADG(F/G). E1 diminished the activities of catalase and glutathione peroxidase, while it increased the content of interleukin, tumor necrosis factor-alpha, and reactive oxygen species (ROS) (*p* < 0.05). Cecal microbiome showed that the abundance of *Firmicutes* and *Actinobacteria* in E1 was significantly lower than in E2 (*p* = 0.029, *p* = 0.002), whereas *Bacteroidetes* was higher (*p* = 0.005). E1 increased the genera *Ruminococcaceae-UCG-004*, *Acinetobacter*, and *Rikenellaceae_RC9_gut_group*. Meanwhile, cecal metabolome showed that formyl-5-hydroxykynurenamine, chorismate, 3-sulfinoalanine, and 3-isopropylmalate were upregulated in E1, while brassinolide was downregulated. The altered metabolites were mainly enriched in metabolic pathways related to energy metabolism and metabolism to mitigate oxidative stress in the meat donkeys, such as tryptophan metabolism, brassinosteroid biosynthesis metabolism, etc. In conclusion, low-energy diets resulted in a negative energy balance in meat donkeys, resulting in more nutrients being oxidized to provide energy, inducing oxidative stress, and thereby leading to decreasing growth. The reduction in meat donkey growth from low-energy diets was related to changes in cecum microbiota and metabolites.

## 1. Introduction

Upon domestication, donkeys (*Equus asinus*) were primarily used as draft animals, but in recent years, they have been used to provide leather, milk, and meat [1]. Donkey meat, which has high contents of crude protein, essential amino acids, and unsaturated fatty acids, but low total fat, cholesterol, and calories, is greatly appreciated by consumers [2]. However, current sloppy feeding management and an insufficient nutritional supply lead to the slow growth of meat donkeys, so it is necessary to improve the growth performance of meat donkeys. Dietary energy directly contributes to the growth performance and meat production of meat animals. Some researchers have shown that low dietary energy levels can reduce daily weight gain and increase the feed-to-gain ratio (F/G) in cattle and lambs [3,4]. However, at present, few studies on the energy nutrition of donkeys are available. Limited research has reported that lower dietary energy levels reduced the growth of 9-month-old donkeys, which was probably related to the upregulation of growth-related metabolic pathways in the rectum [5]. However, the exact mechanism was not known.

Nutrient digestibility is an influential factor of growth performance. Equine animals have a strong microbial fermentation system in the hindgut, including the cecum and colon, and in particular, cecum microbial fermentation is dominant, providing a site for equine fiber digestion. The volatile fatty acids (VFAs) produced by fiber fermentation provide 60–70% of energy to equine animals. The cecum microbial fermentation in equine animals can improve animal immunity, promote nutrient digestion and absorption, and increase the intestinal protective barrier [6]. Feeding patterns and dietary energy levels affect growth, nutrient digestion, antioxidant capacity, and inflammatory factor levels in donkeys by influencing cecal or rectal microbes [7,8]. In addition, oxidative stress adversely affects performance parameters, such as the apparent digestibility of nutrients and growth performance of donkeys [9]. In the event of a shortage of adequate energy from diet, the organism bridges the energy deficit by the oxidative process of non-esterified fatty acids (NEFAs) produced from body fat. The β-oxidation of NEFA produces large amounts of reactive oxygen species (ROS), and excessive free radicals break the balance between oxidation and antioxidant systems, potentially leading to oxidative stress. Oxidative stress is defined as the imbalance between an increase in ROS levels and the cell’s ability to neutralize them via the antioxidant system and the repair/turnover mechanism [10].

Furthermore, several studies have shown that the mechanisms regulating animal growth performance, lactation performance, and antioxidant function are associated with the “gut microbe–metabolite” axis [11,12]. In the current study, it was hypothesized that lower-energy-level diets decrease the growth performance of meat donkeys by altering microorganisms and metabolites in the cecum and causing oxidative stress. Therefore, this study aimed to investigate the effect of dietary energy levels on meat donkeys’ growth performance and explore potential mechanisms in terms of nutrient digestibility, cecal fermentation, and antioxidants.

## 2. Materials and Methods

### 2.1. Experimental Design, Diet, and Feeding Management

A single-factor completely randomized design was used. Twelve meat donkeys (male) aged 1 year with a similar weight (150 ± 25 kg) were divided into two treatment groups, low-energy group (E1) and high-energy group (E2), which were based on the nutritional needs of miniature horses [13]. The experiment was divided into a 10-day pre-trial period and into a 135-day trial period. Donkeys in the trial periods were fed diets with digestible energy values (in dry matter) of 12.08 and 13.38 MJ/kg (pre-fattening, PF, 1–45 d), 13.01 and 14.27 MJ/kg (mid-fattening, MF, 46–90 d), and 13.54 and 14.93 MJ/kg (late fattening, LF, 91–135 d). The treatment diets were offered to donkeys twice daily at 07:00 and 14:00. The donkeys were placed into individual pens with a separate feeder, and water was supplied ad libitum. Deworming was carried out before the adaptation period, and the pens were cleaned and disinfected regularly. Donkeys in each group in the pre-trial period were fed low-energy diets, and the rest of the feeding management practices were the same as those in the trial period. The dietary composition and nutrient level are shown in Table 1.

### 2.2. Sample Collection

Samples of feed were collected at the beginning of the trial and stored at −20 °C for chemical analysis. During the late-fattening period (127–133 d) for 7 consecutive days, fecal samples were collected 3 times a day using the rectal collection method [14], with a minimum of 200 g each time, and all samples from each donkey were mixed thoroughly. Feed and fecal samples were dried in a forced-air oven at 65 °C for 72 h, and then ground through a 1 mm screen.

Blood samples were collected into test tubes (Corning Incorporated Costar, Corning, NY, USA) from the jugular veins of all donkeys before morning feeding at the end of the late-fattening period, and then centrifuged at 2500× *g* for 15 min to separate the serum. The serum was stored at −20 °C and determined for biochemical parameters and antioxidant and immune indicators.

At the end of the experiment, all donkeys were slaughtered by exsanguination. Before slaughter, the animals were fasted for 24 h and prohibited from drinking water for 2 h. The cecum was dissected, and its contents and mucosa were collected and snap-frozen in liquid N2 and stored at −80 °C. Cecal contents were used for microbial diversity and metabolome analyses. Cecal mucosa homogenates were prepared in ice-cold physiological saline (Hebei Kexing Pharmaceutical Co., Ltd., Shijiazhuang, Hebei, China). The homogenate was centrifuged at 5000× *g*, 4 °C, for 15 min. The supernatant obtained was used for determining antioxidant and immune indicators. We collected cecal fluid and stored it at −20 °C for the determination of VFAs. Meanwhile, the duodenum, jejunum, and ileum were collected for the determination of digestive enzyme activity.

### 2.3. Growth Performance

Before the start of the late-fattening stage, all donkeys were weighed on an empty stomach in the morning, and their initial body weight was recorded. After that, the average daily gain (ADG) and total weight gain (TWG) were calculated by weighing at the end of the experiment. During the experiment, the dry matter intake (DMI) was recorded in replicates, and the F/G ratio was calculated. The formulas are as follows:

TWG = End weight − start weight

ADG = TWG/test days;

F/G = DMI/ADG.

### 2.4. Nutrient Digestibility

All feed and fecal samples were evaluated for the subsequent analyses of crude protein (CP method 954.01) and ether extract (EE method 920.39), according to the Association of Official Analytical Chemists (AOAC) [15]. Neutral detergent fiber (NDF) and acid detergent fiber (ADF) were determined according to the methods described by Van Soest et al. [16]. Acid insoluble ash (AIA) was used to determine the apparent total tract digestibility (ATTD) of a certain nutrient according to the description of Keulen et al. [17] using the following formula:

ATTD (%) = 100 − [(A1 × B)/(A × B1)] × 100.

where A is the content of a nutrient in the diet (%), A1 is the content of the same nutrient in the feces (%), B is the content of AIA in the diet (%), and B1 is the content of AIA in the feces (%).

### 2.5. Serum Biochemical Indices and Antioxidants and Immune Cytokines in the Serum and Cecum

Concentrations of glucose (GLU), cholesterol (CHO), urea nitrogen (UREA), and NEFA in serum were determined by using an automatic biochemical analyzer (7020 Automatic Analyzer, 713-0002, HITACHI, Tokyo, Japan). The activities of catalase (CAT, Visible light, CAS: A007-1-1), glutathione peroxidase (GPx, Colorimetric method, CAS: A005-1-2), and total superoxide dismutase (T-SOD, Hydroxylamine method, CAS: A001-1-2), and malondialdehyde (MDA, TBA method, CAS: A003-1-2) concentration in the serum and cecum were determined using commercial antioxidant kits (Nanjing Jiancheng Bioengineering Institute of China, Nanjing, China), according to the manufacturer’s protocols. The concentrations of interleukin (IL)-1β, IL-2, IL-6, IL-4, and IL-10, and tumor necrosis factor-alpha (TNF-α), nitric oxide (NO), and ROS were assayed using commercial ELISA kits from the Beijing Sinouk Institute of Biological Technology, Beijing, China.

### 2.6. Cecal VFAs

VFAs include acetate, propionate, butyrate, isobutyrate, isovalerate, and valerate, which were determined by gas chromatography according to Xie et al. [18], with some modifications. After thawing at 4 °C, cecal fluids were mixed and then centrifuged at 3500× *g* for 15 min at 4 °C, and 0.2 mL of metaphosphoric acid solution (250 g/L) containing 2 g/L 2-ethyl butyrate was added to 1.2 mL of supernatant. The mix was vortexed and centrifuged at 10,000× *g* for 10 min at 4 °C, and 1 μL of supernatants was injected into the gas chromatography apparatus (GC-2014ATFSPL, Shimadzu, Kyoto, Japan; film thickness of the capillary column: 60 m × 0.25 mm × 0.50 μm; column temperature: 180 °C; injector temperature: 220 °C; and detector temperature: 250 °C) for analysis.

### 2.7. Digestive Enzyme Activity of the Small Intestine

The duodenum, jejunum, and ileum were opened longitudinally to scrape off the mucosa, which was homogenized and centrifuged, and the supernatant was obtained for the determination of digestive enzyme activities as follows. We determined the digestive enzyme activities of α-amylase, chymotrypsin, trypsin, and lipase in the duodenum, jejunum, and ileum. The activities of α-amylase (CAS: C016-1-1) and lipase (CAS: A054-1-1) were assayed using the colorimetric method with commercial kits (Nanjing Jiancheng Bioengineering Institute of China, Nanjing, China), according to the manufacturer’s protocols. The activities of chymotrypsin (CAS: CK-E73343) and trypsin (CAS: CK-E73341) were assayed using commercial ELISA kits from R&D Systems (Minneapolis, MN, USA).

### 2.8. Cecal Microbiota and Microorganism Analyses

Total microbial genomic DNA was extracted from cecal samples using the E.Z.N.A.^®^ soil DNA Kit (Omega Bio-tek, Norcross, GA, USA), according to the manufacturer’s instructions. The quality and concentration of DNA were determined by 1.0% agarose gel electrophoresis. The variable region of the bacterial 16S rRNA V3–V4 gene was amplified using the primers 338F (5′-ACTCCTACGGGAGGCAGCAG-3′) and 806R (5′-GGACTACHVGGGTWTCTAAT-3′) with a unique barcode. The PCR product was extracted from 2% agarose gel and purified using the AxyPrep DNA Gel Extraction Kit (Axygen Biosciences, Union City, CA, USA), according to the manufacturer’s instructions, and quantified using the Quantus™ Fluorometer (Promega, Madison, WI, USA). Purified amplicons were pooled in equimolar amounts and paired-end sequenced on an Illumina MiSeq PE300 platform (Illumina, San Diego, CA, USA), according to the standard protocols by Majorbio Bio-Pharm Technology Co., Ltd. (Shanghai, China). Bioinformatic analysis of gut microbiota was carried out using the Majorbio Cloud platform (https://cloud.majorbio.com). Based on the OTU information, rarefaction curves were calculated with Mothur v1.30. A total of 12 cecum samples was analyzed using the LC-MS platform (Thermo Fisher Scientific UHPLC-Q Exactive HF-X system, Waltham, MA, USA). To obtain information about the reproducibility of the system, quality control (QC) samples were injected into every 6 samples analyzed throughout the analysis. After mass spectrometry detection was completed, the raw data of LC/MS were preprocessed using Progenesis QI (Waters Corporation, Milford, CT, USA, version 3.0) software. At the same time, the metabolites were searched and identified, and the main databases used were the HMDB (http://www.hmdb.ca), Metlin (https://metlin.scripps.edu), and Majorbio Database. The data following the database search were uploaded onto the Majorbio cloud platform (https://cloud.majorbio.com) for data analysis.

### 2.9. Statistical Analysis

Growth performance was analyzed using the PROC MIXED procedure of SAS (version 8.1, SAS Institute Inc., Cary, NC, USA). The statistical model was used, with the dietary energy treatment (E1, E2) and the fattening stages (PF, MF, and LF) considered fixed effects, and the donkey presenting a crossover design and the crossover period were considered a random effect. Student’s *t*-test was used to analyze nutrient digestibility, serum biochemical indices, cecal VFAs, digestive enzyme activity of the small intestine, alpha diversity indexes, and differential microorganisms at the phylum level. Statistical significance was set at *p* < 0.05. Linear discriminant analysis (LDA) coupled with effect size measurement (LEfSe) analysis was conducted to search for statistically different microbial groups between groups at the genus, and the LDA score was 2. Spearman correlation was used to correlate growth performance with the result of LEfSe bacterial genera using R (heatmap package, version 3.3.1). Only correlations with *p* < 0.05 for the linear model were considered significant. PCoA was performed using the Bray–Curtis distance with R. Nonparametric multivariate analysis of variance (Adonis) was used to assess the significance of differences in bacterial community structures among groups. Statistical significance was set at *p* < 0.05.

## 3. Results

### 3.1. Growth Performance, Nutrient Digestibility, Serum Biochemical Indices, and Cecal VFAs

E2 differentially increased BW and ADG and decreased the F/G ratio (Table 2). As shown in Table 3, E2 significantly increases the digestibility of CP, EE, ADF, and NDF; body weight (BW); ADG; and CHO compared to E1 (*p* < 0.05), whereas the concentrations of the F/G ratio and NEFA decrease. And UREA in E1 tends to be higher than that in E2. However, there are no significant differences in the DMI and GLU between the two groups. Table 4 shows that donkeys in E2 have a lower concentration of total VFAs, acetate, propionate, butyrate, isobutyrate, isovalerate, valerate, and A/P ratio (*p* < 0.05).

### 3.2. Digestive Enzyme Activity of the Small Intestine

As shown in Table 5, E1 decreases α-amylase activity in the jejunum and ileum as well as trypsin activity and lipase activity in the duodenum, jejunum, and ileum (*p* < 0.05).

### 3.3. Serum Antioxidant Activities and Immune Signaling Molecule Levels

Table 6 shows the antioxidant enzyme activities, MDA concentration, and immune signaling molecule in serum. E2 had a greater value of activities of CAT and GPx, as well as the concentrations of IL-4 and IL-10 in serum (*p* < 0.05). On the contrary, E2 significantly reduced the concentration of MDA, IL-1β, IL-2, IL-6, and TNF-α, and ROS activity (*p* < 0.05).

### 3.4. Cecal Antioxidant Activities and Immune Signaling Molecule Levels

Dietary energy levels also have a remarkable influence on antioxidant enzyme activities, MDA concentration, and immune signaling molecules in the cecum (Table 7). E2 elevated activities of CAT and T-SOD and the concentration of IL-4 and IL-10 in serum (*p* < 0.05), while decreased the concentration of IL-1β, IL-2, IL-6, and TNF-α, and ROS activity (*p* < 0.05).

### 3.5. Cecal Bacterial Diversity

#### 3.5.1. Rarefaction Curves, Alpha Diversity, and PCoA

The dilution curve tends to be flat with the increase in the sample size, indicating that the amount of sequencing data is large enough to ensure the reliability of the data analysis (Figure S1A). There were no significant differences in the cecal contents of the two groups, but the samples between the two groups were separated. The inter-group differences in each group were greater than the intra-group differences (R > 0), indicating the significance of this experimental grouping (Figure S1B). E2 tended to increase the Chao index, but there were no significant differences in Sobs, Ace, Shannon, and Simpson, and the value of coverage was 0.99, which indicated that the sequencing depth could reflect the diversity of microorganisms in the samples (Table S1).

#### 3.5.2. Microbial Composition

**Figure 1:**
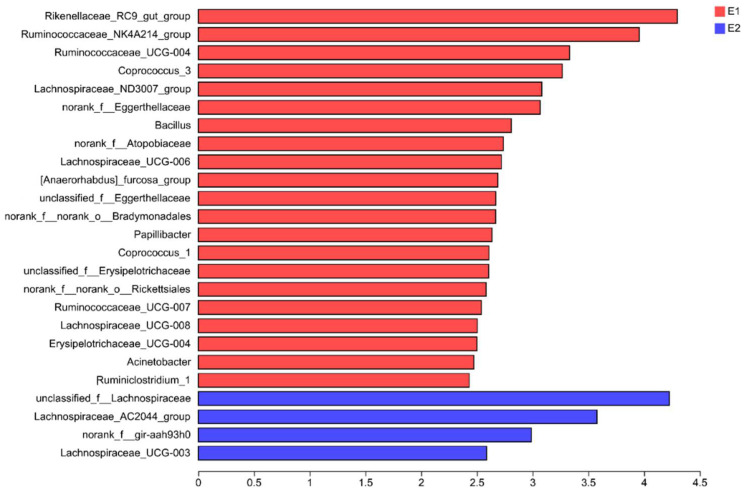
Effects of dietary energy level on the composition of microflora at the genus level. Each color represents one treatment: The red curves represent donkeys fed a low-energy (E1) diet and the blue curves represent donkeys fed a high-energy (E2) diet.

As can be seen from Table 8, the abundance of *Firmicutes* and *Actinobacteria* in E2 is significantly higher than that in E1 (*p* = 0.029, *p* = 0.002), whereas Bacteroidetes are lower (*p* = 0.005), but dietary energy level has no significant effect on the abundance of *Spirochaetes*, *Tenericutes*, and *Proteobacteria*. LEfSe analysis (LDA threshold = 2, Figure 1) showed that 21 bacterial genera (the red part), including *Rikenellaceae_RC9_gut_group*, *Ruminococcaceae_NK4A214_group*, and *Ruminococcaceae_UCG_004*, were significantly enriched in E1. *Unclassified_f_Lachnospiraceae*, *Lachnospiraceae_AC2044_group*, *norank_f_gir_aah93h0*, and *Lachnospiraceae_UCG_003* were significantly enriched in E2 (the blue part).

### 3.6. Cecal Metabolome

#### 3.6.1. Analysis by PCA and OPLS-DA

To compare the distribution of the cecal metabolites of the two groups, PCA and OPLS-DA were conducted (Figure S2). As shown, the metabolites in cecal samples were clearly separated in a pairwise comparison between the two treatment groups, and there was no overlap in the positive and negative ion modes (Figure S2A–D), and, except for the negative ion score for the E2 vs. E1 group (Figure S2A), the samples were basically in the 95% confidence interval. R2Y values in the positive and negative ion modes were 0.979 and 0.995, respectively, which illustrated the model can simulate the real situation of sample data well (Figure S2E,F).

#### 3.6.2. Differential Metabolites

**Figure 2:**
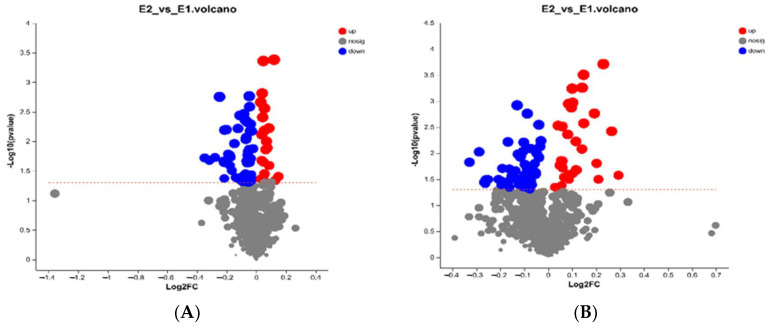
(**A**) Differential metabolites in E2 vs. E1 in Pos. (**B**) Differential metabolites in E2 vs. E1 in Neg. The red curves represent upregulated metabolites, the blue represents downregulated metabolites, and the gray represents no change. E1 = low-energy group; E2 = high-energy group.

Student’s *t*-test and fold difference analysis were performed. The selection of significantly different metabolites was determined based on the variable importance in the projection (VIP) obtained by the OPLS-DA model and the *p*-value of Student’s *t*-test, and the metabolites with VIP > 1, *p* < 0.05 were significantly different metabolites. A total of 159 differential metabolites was screened, among which 51 metabolites were upregulated, while 108 were downregulated (Figure 2A,B). Most of the differential metabolites enriched were associated with lipids and lipid-like molecules, phenylpropanoids and polyketides, organic oxygen compounds, and organic acids and derivatives (Table 9). 

#### 3.6.3. Differential Pathways

**Figure 3:**
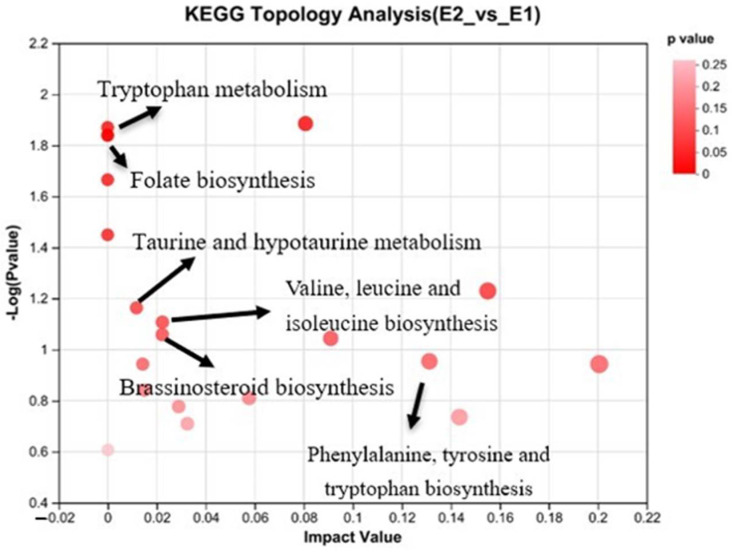
Pathway enrichment analysis was performed using the significantly different metabolites for E2 vs. E1. E1 = low-energy group; E2 = high-energy group. The red bubbles represent metabolic pathways.

Differential metabolites among two groups were summarized and mapped into their biochemical pathways through metabolic enrichment and pathway analysis based on a database search (KEGG, http://www.genome.jp/kegg/pathway.html, accessed on 4 June 2023). After analysis by Scipy.stats (Python package, https://www.docs.scipy.org/doc/scipy/.html, accessed on 4 June 2023), a total of 25 pathways was enriched, among which six pathways were significantly enriched. Differential metabolism is shown in Figure 3 and Table 10, including tryptophan metabolism; folate biosynthesis; taurine and hypotaurine metabolism; ubiquinone and terpenoid–quinone biosynthesis; brassinosteroid biosynthesis; phenylalanine, tyrosine, and tryptophan biosynthesis; starch and sucrose metabolism; and galactose metabolism.

### 3.7. Spearman Correlation Analysis

#### 3.7.1. Spearman Correlation Analysis Between Cecal Discrepant Bacteria (Figure 2) and Nutrient Digestibility, Growth Performance and Serum Biochemical Parameters

**Figure 4:**
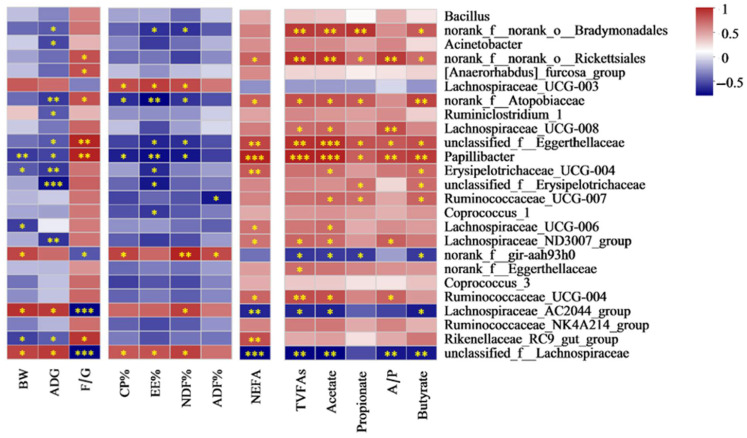
Correlation analysis of cecal content bacteria and growth performance, nutrient digestibility, NEFA (non-esterified fatty acid) concentration, cecal volatile fatty acids. Red color: positive correlation; blue color: negative correlation. * *p* < 0.05; ** *p* < 0.01; *** *p* < 0.001. Abbreviations: BW = body weight; ADG = average daily gain; F/G = the ratio of dry matter intake to ADG. CP% = the digestibility of crude protein; EE% = the digestibility of ether extract; ADF% = the digestibility of acid detergent fiber; NDF% = the digestibility of acid detergent fiber. TVFAs = total volatile fatty acids; A/P, the ratio of acetate to propionate.

Spearman correlation analysis was conducted on the growth performance, with the result of LEfSe bacteria at the genus level (Figure 4). The threshold |R| > 0.5 is considered a significant Spearman correlation. *Norank_f__norank_o__Bradymonadales* was negatively correlated with the digestibility of EE and NDF, ADG. Acinetobacter was negatively correlated to ADG. *Norank_f__norank_o__Rickettsiales* was positively connected to the F/G ratio and NEFA concentration. *[Anaerorhabdus]_furcosa_group* was positively connected to the F/G ratio. There was a negative relation between *Lachnospiraceae-UCG-003* and the digestibility of EE, CP, and NDF. *Norank_f__Atopobiaceae* was negatively correlated with the digestibility of EE, CP and NDF, ADG, whereas it was positively correlated with the F/G ratio. *Ruminiclostridium_1* was negatively connected to ADG. *Unclassified_f__Eggerthellaceae* and *Papillibacter* were negatively correlated with the digestibility of EE and NDF, ADG, whereas they were positively correlated with the F/G ratio and the concentration of NEFAs. Meanwhile, Papillibacter was negatively correlated with the digestibility of CP and BW. *Erysipelotrichaceae_UCG_004* was negatively correlated with the digestibility of EE, BW, and ADG, whereas the concentration of NEFA Acetate and Butyrate was the opposite. There was a negative relation between *unclassified_f__Erysipelotrichaceae* and the digestibility of EE, ADG. *Ruminococcaceae_UCG_007* was negatively correlated with the digestibility of ADF, while positively correlated with ADF, Acetate and Butyrate. There was a negative relation between *Coprococcus_1*, *Lachnospiraceae_UCG_006*, *unclassified_f__Eggerthellaceae*, and digestibility of EE and BW, separately. *Lachnospiraceae_ND3007_group* was negatively correlated with ADG, while positively correlated with NEFA, TVFAs and Acetate. *Norank_f__gir_aah93h0* was positively correlated with the digestibility of ADF, CP, NDF, and BW, whereas the F/G ratio was the opposite. There was a positive relationship between *Ruminococcaceae-UCG-004* and the concentration of NEFAs. *Lachnospiraceae_AC2044_group* and *unclassified_f__Lachnospiraceae* were negatively correlated with the F/G ratio and the concentration of NEFAs, while they were positively correlated with the digestibility of NDF, BW, and ADG. Meanwhile, *unclassified_f__Lachnospiraceae* was positively correlated with the digestibility of EE and CP. *Rikenellaceae_RC9_gut_group* was positively related to the F/G ratio and NEFA concentration, whereas BW and ADG showed the opposite. The concentration of VFAs and the A/P ratio were negatively correlated with *Lachnospiraceae-UCG-003*, *Norank_f__gir_aah93h0*, *Lachnospiraceae_AC2044_group*, and *unclassified_f__Lachnospiraceae*, while they were positively correlated with other bacteria.

#### 3.7.2. Spearman Correlation Analysis of Cecal Differential Metabolites and Nutrient Digestibility, Growth Performance, and Serum Biochemical Parameters

**Figure 5:**
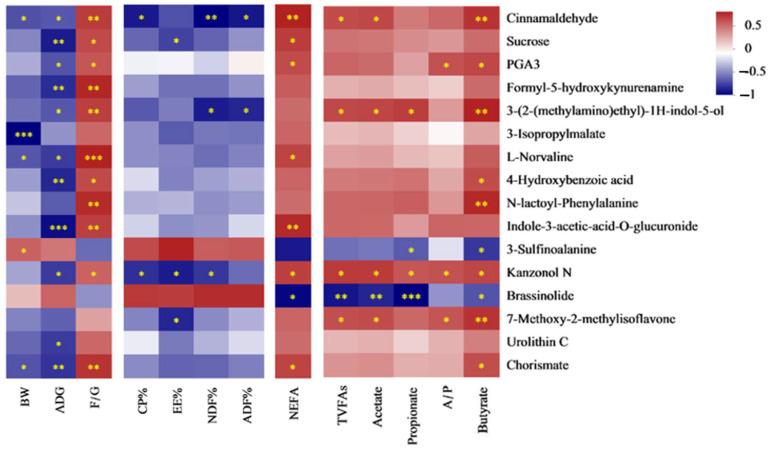
Correlation analysis of cecal differential metabolites and growth performance, nutrient digestibility, NEFA (non-esterified fatty acid) concentration, and cecal volatile fatty acids. Red color: positive correlation; blue color: negative correlation. * *p* < 0.05; ** *p* < 0.01; *** *p* < 0.001. Abbreviations: BW = body weight; ADG = average daily gain; F/G = the ratio of dry matter intake to ADG. CP% = the digestibility of crude protein; EE% = the digestibility of ether extract; ADF% = the digestibility of acid detergent fiber; NDF% = the digestibility of acid detergent fiber. TVFAs = total volatile fatty acids; A/P, the ratio of acetate to propionate.

The threshold |R| > 0.5 is considered a significant Spearman correlation. Some results are shown in Figure 5. PGA3 and Indole-3-acetic-acid-O-glucuronide (IAA) were negatively connected to ADG, while they were positively connected to the F/G ratio and NEFAs. L-Norvaline was negatively connected to ADG and BW, while showing a positive relationship with the F/G ratio and the concentration of NEFAs. There was a negative relationship between brassinolide and the concentration of NEFAs. Cinnamaldehyde was negatively associated with ADG, BW, and the digestibility of CP, ADF, and NDF, while showing a positive connection to the F/G ratio and the concentration of NEFAs. Sucrose and Kanzonol N had a negative relationship with ADG and the digestibility of EE, but showed a positive connection to the F/G ratio and the concentration of NEFAs. And, also, there was a negative relationship between Kanzonol N and the digestibility of CP and NDF. Formyl-5-hydroxykynurenamine (f5-HK) was negatively correlated in ADG, while exhibiting a positive correlation in the F/G ratio. Chorismate was negatively connected to ADG and BW, but showed a positive relationship with the F/G ratio and the concentration of NEFAs. 3-Isopropylmalate was negatively associated with BW. 7-Methoxy-2-methylisoflavone showed a negative correlation to the digestibility of EE. Urolithin C and 4-Hydroxybenzoic acid were negatively correlated with ADG. Finally, 4-Hydroxybenzoic acid was positively correlated with the F/G ratio. There was a negative relationship between N-lactoyl-Phenylalanine and the F/G ratio. 3-(2-(methylamino)ethyl)-1H-indol-5-ol was negatively connected to ADG, NDF%, and ADF%, while it had a positive relationship with the F/G ratio. 3-Sulfinoalanine and brassinolide were negatively associated with VFA concentration and the A/P ratio, while other metabolites showed the opposite trend.

#### 3.7.3. Spearman Correlation Analysis of Cecal Discrepant Bacteria and Antioxidant and Immune Indicators in the Serum and Cecum

**Figure 6:**
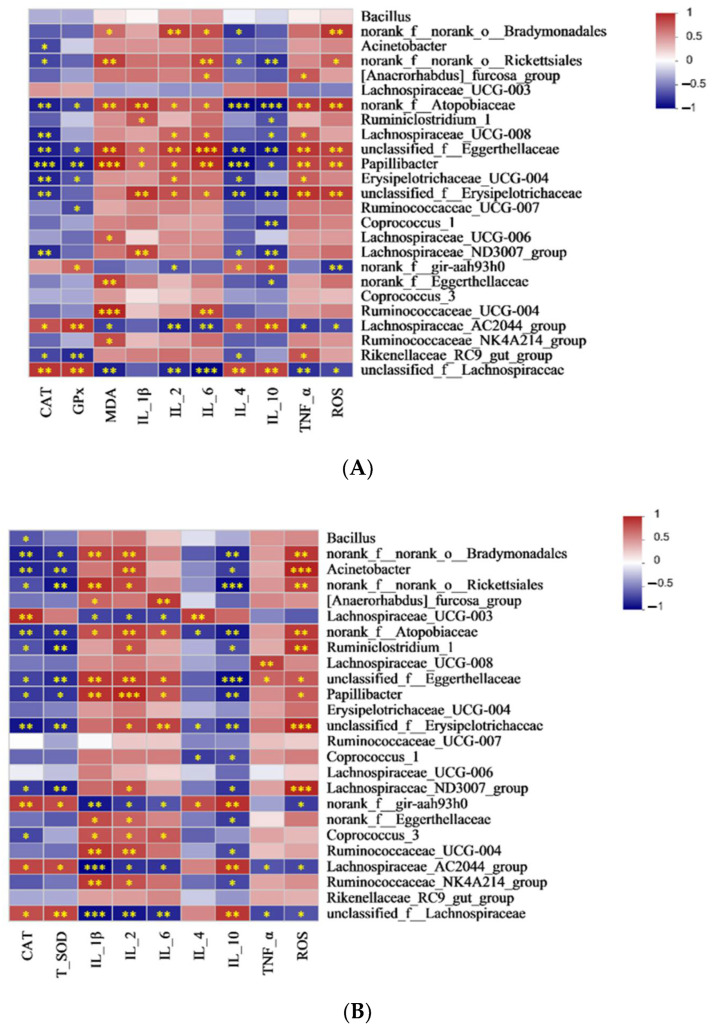
Correlation analysis of cecal content bacteria and (**A**) serum antioxidant and immune indicators; (**B**) cecum antioxidant and immune indicators. Red color: positive correlation; blue color: negative correlation. * *p* <0.05; ** *p* < 0.01; *** *p* < 0.001. Abbreviations: CAT = catalase; GPx = glutathione peroxidase; T-SOD = total superoxide dismutase; MDA = malondialdehyde; IL = interleukin; TNF-α = tumor necrosis factor-alpha; NO = nitric oxide; and ROS = reactive oxygen species.

Cecal microorganisms are closely related to enzyme activities and immune factors in the serum and cecum (Figure 6). Among them, in the serum, *Acinetobacter* was negatively associated with CAT. Ruminococcaceae_UCG_004 was positively related to MDA and IL-6. *Rikenellaceae_RC9_gut_group* was negatively correlated with GPx, CAT, and IL-4, while it was positively correlated with TNF-α (Figure 6A). As shown in Figure 6B, Acinetobacter is negatively associated with CAT, T-SOD, and IL-10, while it is positively correlated with IL-2 and ROS in the cecum. *Ruminococcaceae_UCG_004* was positively correlated with IL-1β and IL-2, whereas it was negatively associated to IL-10.

#### 3.7.4. Spearman Correlation Analysis of Cecal Differential Metabolites and Antioxidant and Immune Indicators in the Serum and Cecum

**Figure 7:**
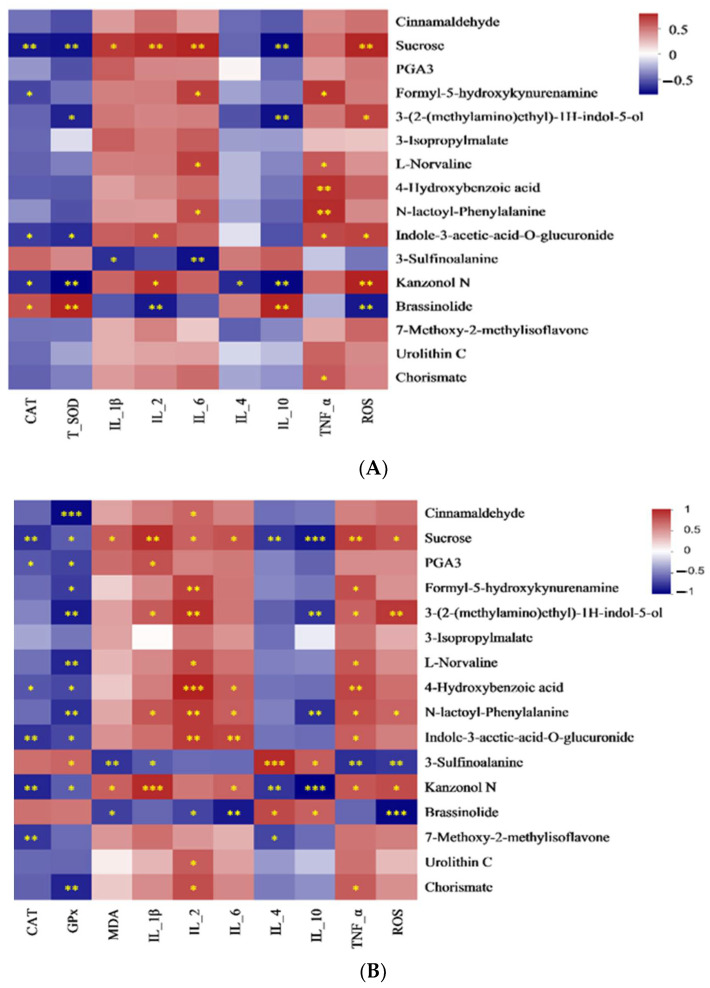
Correlation analysis of cecal content bacteria and (**A**) serum antioxidant and immune indicators; (**B**) cecum antioxidant and immune indicators. Red color: positive correlation; blue color: negative correlation. * *p* < 0.05; ** *p* < 0.01; *** *p* < 0.001. Abbreviations: CAT = catalase; GPx = glutathione peroxidase; T-SOD = total superoxide dismutase; MDA = malondialdehyde; IL = interleukin; TNF-α = tumor necrosis factor-alpha; NO = nitric oxide; and ROS = reactive oxygen species.

Figure 7 shows a strong correlation between metabolites and antioxidant and immune indicators in the serum and cecum. F5-HK and chorismate are negatively connected to GPx, while they are positively connected to IL-2 and TNF-α. 3-Sulfinoalanine is positively connected to MDA, IL-1β, and ROS, but negatively associated with IL-4 and IL-10, while the relationship between brassinolide and the above-mentioned indicators shows the opposite trend. In addition, 3-sulfinoalanine is negatively correlated with GPx, but was positively connected to TNF-α. Brassinolide shows a negative correlation with IL-2 and IL-6 (Figure 7A). F5-HK was positively correlated with IL-6 and TNF-α, and there was a negative relationship between F5-HK and CAT. 3-Sulfinoalanine was negatively correlated with IL-1β and IL-6. Brassinolide was positively connected to CAT, T-SOD, and IL-10, but was negatively connected to IL-2 and ROS. Chorismate was positively connected to TNF-α.

#### 3.7.5. Spearman Correlation Analysis of Cecal Differential Metabolites and Discrepant Bacteria

**Figure 8:**
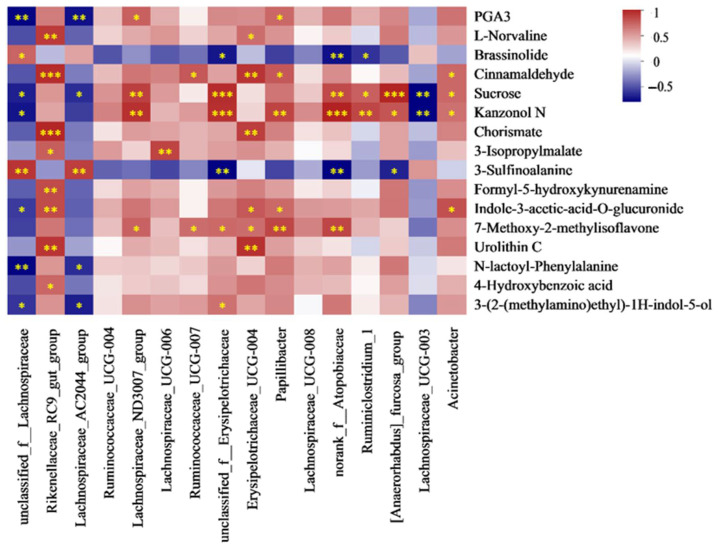
Correlation analysis of cecal differential metabolites and bacteria. Red color: positive correlation; * *p* < 0.05; ** *p* < 0.01; *** *p* < 0.001.

To further clarify the association between cecal microbiota and metabolites, a correlation analysis of the differential cecal bacteria and metabolites was performed using the Spearman test (Figure 8). It showed that PGA3 was negatively connected to *unclassified_f__Lachnospiraceae* and *Lachnospiraceae_AC2044_group*, while it was positively connected to *Lachnospiraceae_UCG_008* and *Lachnospiraceae_ND3007_group*. L-Norvaline and 4-Hydroxybenzoic acid were positively correlated with *Rikenellaceae_RC9_gut_group*, in addition to L-Norvaline. There was a positive relationship between brassinolide and *unclassified_f__Lachnospiraceae*, while there was a negative relationship with *unclassified_f__Erysipelotrichaceae*, *norank_f_Atopobiaceae*, and *Ruminiclostridium_1*. There was a positive relationship between Cinnamaldehyde and *Rikenellaceae_RC9_gut_group*, *Erysipelotrichaceae_UCG_004*, *Lachnospiraceae_UCG_007*, *Lachnospiraceae_UCG_008*, and *Acinetobacter*. Sucrose and kanzonol N had a negative relationship with *unclassified_f__Lachnospiraceae* and *Lachnospiraceae_ND3007_group*, but *Ruminococcaceae_UCG_007*, *unclassified_f__Erysipelotrichaceae*, *Papillibacter*, *Lachnospiraceae_UCG_003*, *norank_f__norank_o_Rickettsiales*, and *Acinetobacter* showed the opposite trend. Meanwhile, there was a negative and positive relationship between the two metabolites and *Lachnospiraceae_AC2044_group* and *Papillibacter*, separately. Chorismate showed a positive correlation with *Rikenellaceae_RC9_gut_group* and *Erysipelotrichaceae_UCG_004*, and 3_Isopropylmalate was positively correlated with *Rikenellaceae_RC9_gut_group* and *Lachnospiraceae_UCG_006*. 3-Sulfinoalanine was positively correlated with *unclassified_f_Lachnospiraceae* and *Lachnospiraceae_AC2044_group*, while it was negatively correlated with *unclassified_f__Erysipelotrichaceae*, *norank_f_Atopobiaceae* and *[Anaerorhabdus]_furcosa_group*. F5-HK was positively correlated with *Rikenellaceae_RC9_gut_group*. IAA_O_glucuronide was negatively correlated with *unclassified_f_Lachnospiraceae*, while exhibiting a positive correlation with *Rikenellaceae_RC9_gut_group*, *Erysipelotrichaceae_UCG_004*, *Papillibacter*, and *Acinetobacter*. There was a positive relationship between 7_Methoxy_2_methylisoflavone and *Lachnospiraceae_ND3007_group*, *Ruminococcaceae_UCG_007*, *Papillibacter*, *unclassified_f_Erysipelotrichaceae*, *Erysipelotrichaceae_UCG_004*, and *norank_f_Atopobiaceae*. Urolithin C showed positive connections to *Rikenellaceae_RC9_gut_group* and *Erysipelotrichaceae_UCG_004*. There was a negative connection between N_lactoyl_Phenylalanine, 3_(2_(methylamino)ethyl)_1H_indol_5_ol, *unclassified_f_Lachnospiraceae*, and *Lachnospiraceae_AC2044_group*. Meanwhile, 3_(2_(methylamino)ethyl)_1H_indol_5_ol was positively correlated with *unclassified_f_Erysipelotrichaceae*.

## 4. Discussion

In the present study, it was concluded that a low-energy diet decreased the growth performance of meat donkeys, which was reflected in the lower body weight and ADG and higher F_G ratio. Similar conclusions were determined in the research on sheep [19,20]. Similarly, Zhang et al. demonstrated that feeding LE (DE = 10.43 MJ/kg) to donkeys reduced their growth performance in terms of ADG and feed conversion efficiency [5]. However, the DE values in the current study were different from the abovementioned study. It was conducted on donkeys at 4 months of age, and the meat donkeys in this trial were around 12 months of age, which may account for the differences in the results of the study on energy nutritional requirements. The digestion of nutrients has a direct impact on ADG. Moreover, the lower metabolism reduced the digestibility of CP, EE, NDF, and ADF in female donkeys [21]. In the current study, the digestibility of CP, EE, NDF, and ADF was declined in E1, which supported the results for growth performance. Moreover, E1 reduced the digestive enzyme activity of the small intestine, which also supported the abovementioned perspective. Furthermore, bacterial diversity and metabolites in the cecum are closely related to nutrient digestion and further affect growth performance. Firmicutes have a strong ability to depolymerize dietary fibers [22], and a high Firmicutes/Bacteroidetes ratio has been directly linked to weight gain [23]. In the current study, donkeys fed with low-energy diets had lower Firmicutes, resulting in a lower *Firmicutes*/*Bacteroidetes* ratio in the cecum. A negative energy balance is reflected by increased NEFA concentrations [24]. Increasing NEFA concentrations indicate that the body lacks energy and will promote steatolysis to energize itself. We detected donkeys fed with low dietary energy had greater serum NEFAs, which may lead to a reduction in the growth performance, resulting in a lack of energy to meet basal metabolism requirements. UREA is the main product of protein metabolism, and there was a trend of elevated UREA in E1 in this experiment, suggesting that protein catabolism was accelerated in E1, which may be used to make up for the lack of dietary energy. As a way of compensating for this, it used body fat for energy and, even worse, consumed protein to make up for this shortfall. It was observed that a lower energy level can decrease the deposition of protein [25]. Yerradoddi et al. reported that the energy level of diets influenced the growth performance and N retention of goats [4]. This further explained that low-energy diets may be utilized for energy supply by catabolizing proteins, resulting in lower protein digestibility. In addition, dietary energy level is an important indicator for the regulation of DMI. Ahmad et al. stated that decreasing dietary energy levels in yaks increased DMI [26]. However, other research has indicated that a low-energy diet had no impact on the DMI of donkeys, but reduced their growth [5]. And the present study’s results are confirmed by the abovementioned results, which may be related to the fact that the difference in energy levels between the two groups was not very obvious. Fewer studies on donkeys are available. Only two dietary energy-level models were designed to explore their effects on the growth of meat donkeys in the present experiment, so the results need to be investigated further.

However, to compensate for this energy supply shortage, NEFA was oxidized to produce large amounts of energy, along with the production of ROS. What is worse, ROS also stimulates the release of inflammatory interleukin TNF-α from macrophages. It has been reported that TNF-α regulation depends on ROS stimuli, and TNF-α can also trigger ROS production [27], suggesting the interaction of oxidative stress and inflammation [28]. Shihata et al. [29] showed that oxidative stress enhanced inflammation-related gene expression and increased inflammatory proteins, impairing endothelial function. It was also implied that inflammatory responses are induced by enhancing oxidative stress, and this adversely affects the performance of donkeys [8]. Excessive levels of free radicals and peroxides can cause oxidative stress in the body. Antioxidant enzymes, including CAT, GPx, and T-SOD, scavenge free radicals and peroxides and maintain redox homeostasis [30], as indicated by an increase in antioxidant enzyme activity and a decrease in MDA and ROS concentrations [31]. IL-1β, IL-6, and TNF are three of the most prominent cytokines associated with innate immune response [32]. In the present study, E1 increased the NEFA content in the serum, and MDA, IL-1β, TNF-α. and ROS in the serum and cecum of meat donkeys, which indicated that low-energy diets may put donkeys in a state of oxidative stress, resulting in a reduced growth performance.

A donkey’s hindgut is an immensely enlarged fermentative chamber that includes an extremely abundant and highly complex community of microorganisms, and in particular, a well-developed cecum is the main site for digesting nutrients. Microorganisms and metabolites in the rectums of lactating donkeys were highly correlated with nutrient digestion, serum antioxidant enzyme activities, and inflammatory factor levels [12]. Cecal contents were collected for microbial diversity and metabolome analyses after fasting, in the present study, to explore the effect of dietary energy levels on the growth of meat donkeys. It was concluded that low-dietary-energy levels diminished their growth by altering cecum microflora and metabolites. *Ruminococcaceae_UCG_004* belongs to *Ruminococcaceae*. A large amount of *Ruminococcaceae* may result in a greater release of IL-1β in broiler chickens [33]. In the present study, we found that *Ruminococcaceae_UCG_004* was significantly enriched in E1, which was also positively connected to the concentrations of NEFA, MDA, IL-1β, and IL-6, while it showed a negative relation to ADG. NEFA was negatively connected to brassinolide, whereas it had a positive relation to chorismate. Brassinolide is one of the most biologically active brassinosteroids (BRs) [34]. BRs are one of the novel classes of plant hormones that are polyhydroxysteroids in nature and are essentially involved in plant growth and development [35]. Under stress conditions, BR application substantially improves plant growth by modulating the accumulation of osmoprotectants and antioxidant activity, lowering ROS production and lipid peroxidation [36]. And brassinolide was the differential metabolite in brassinosteroid biosynthesis metabolism that was enriched in the E2 vs. E1 group. Chroismate was a significantly upregulated differential metabolite in the folate metabolic pathway, and high concentrations of folate induce cell death, cytokine release, inflammation, and oxidative stress in male Wistar rats [37]. Consistently, brassinolide was positively connected to CAT and IL-10, but was negatively connected to ROS and NEFAs. Chroismate was negatively connected to GPx, while it was positively connected to TNF-α. The results above suggest that the donkeys in E1 are in a state of oxidative stress, which further leads to a decrease in ADG. Possibly, this was due to the upregulation of the differential metabolite chromate and downregulation of brassinolide due to the increase in the low-energy diet *Ruminococcaceae_UCG_004*, which resulted in the elevation of IL-1β, TNF-α, and ROS, and reduction in CAT, GPx, and IL-10.

*Acinetobacter* baumannii, which induces ROS production, is a major infectious bacterium, whose regulatory role may be related to the absence of CAT and SOD [38,39]. And in the present study, *Acinetobacter*, which was upregulated in E1, was negatively correlated with ADG, CAT, and IL-10, but positively related to ROS. This implied that E1 decreased the growth performance of meat donkeys, maybe due to oxidative stress due to upregulated *Acinetobacter*.

In addition, meat donkeys in the low-energy group compensated for the energy deficit by enhancing energy metabolic pathways, including tryptophan (Trp) metabolism and phenylalanine (phe), tyrosine (tyr), and trp biosynthesis. Guo et al. found that the rectal *Rikenellaceae_RC9_gut_group* in donkeys was negatively correlated with ADG and CP digestibility [8]. Meanwhile, *Rikenellaceae_RC9_gut_group* was upregulated in E1, which was positively connected to F_G and NEFA concentrations, while it was negatively connected to BW and ADG. Serotonin (5- hydroxytryptamine, 5_HT), a metabolite of Trp, is an important gastrointestinal regulatory factor exhibiting a wide range of physiological effects on animals [40]. Formyl-5-hydroxykynurenamine (f5-HK) was a metabolite of 5-HT. An increased level of f5-HK indicates a corresponding increase in 5-HT [41]. Sumara et al. [42] revealed that 5-HT produced during fasting promotes gluconeogenesis that mainly takes place in the liver by enhancing the activity of two key gluconeogenesis rate-limiting enzymes. Furthermore, *Rikenellaceae_RC9_gut_group* was also negatively related to chorismite, which was the differential metabolite enriched in pathways of phe, tyr, and trp biosynthesis. Chorismate was elevated in E1. Chorismate contributes to the synthesis of aromatic amino acids, such as tyr, phe, and trp, in plants and microorganisms [43]. In animals, phe is mainly converted to tyrosine to perform biological actions [44]. Tyr is a precursor for the synthesis of thyroid hormones, norepinephrine, and epinephrine [45], and thyroid hormones promote the breakdown of fats and other substances [46]. In the present study, f5-HK and branchialate were negatively correlated with ADG and positively correlated with the F/G ratio significantly enriched in E1, suggesting that an elevated concentration of f5-HK entering the liver promotes gluconeogenesis as well as the conversion of more branchialate to tyr, and thus more thyroxine. E1 donkeys may increase energy metabolism through the abovementioned pathways, which can compensate for the lack of energy in the diet. However, the mobilization of body fat to provide energy leads to a decrease in the growth performance of donkeys, as less energy is available for growth.

Additionally, valine, leucine, and isoleucine biosynthesis increased in E2 vs. E1, and 3-isopropylmalate, upregulated in E1, was one of the significantly differential metabolites. On the grounds of this biosynthesis, 3-isopropylmalate can be converted to pyruvate and leucine. As we know, pyruvate forms acetyl coenzyme A (acetyl-CoA), entering the tricarboxylic acid (TCA) cycle and releasing a lot of energy. In addition, leucine can transform into acetoacetate, which is also allowed to enter the TCA cycle and yield energy. 3-Isopropylmalate was positively connected to propionate and butyrate. The injection of butyrate into a mouse cecum showed that butyrate entered the cecum as acetyl-CoA [47]. Consistently, the current research showed that E1 increased the concentrations of propionate and butyrate, suggesting that donkeys in E1 obtained energy by the TCA cycle supported by propionate and butyrate, which may be associated with valine, leucine, and isoleucine biosynthesis.

Additionally, in this experiment, an increase in dietary energy levels was matched by a small decrease in fiber. Koh et al. [48] found that an adequate addition of fiber to livestock and poultry diets can maintain the normal structure of the intestinal tract, promote intestinal peristalsis, improve intestinal microbiota, and enhance the organism’s immunity and resistance to disease. Furthermore, there was a considerable difference between the two groups in the ratio of NFC to NDF in the late-fattening-diet group. Considering that the NFC/NDF ratio affects microorganisms in the cecum and their degradation of fiber, a possible cause of microbial changes cannot be ruled out as being related to the NFC/NDF ratio. The colon plays an important function in the fermentation and utilization of nutrients, and a study by Zhang et al. [49] showed that the concentration level in the diet affected the fermentation of goat colonic microorganism. Zhao et al. [50] noted that dietary protein levels affect the abundance and composition of barrow ([Landrace × Yorkshire] × Duroc) colonic microorganisms. However, there is little research on the effect of dietary energy levels on donkey colonic microorganisms. In consideration of the abovementioned research, we will investigate the effect of dietary energy on donkey colonic microorganisms. In the current study, we utilized the Spearman correlation to correlate the measured indicators, such as growth performance-related indicators, cecum VFA concentration, and antioxidant and immune indicators in the serum and cecum, with the differential microorganisms or metabolites obtained from the microbiomics and metabolomics of the cecum in an attempt to find a potential link between them. Although we have not investigated the direct effect of a specific microorganism or metabolite on these indicators, some ideas and data are provided for us to explore the related mechanisms in depth. Due to limited resources, six donkeys per treatment were utilized for the feeding trials. Moreover, employing single pen rearing ensured the reliability and feasibility of the trial results. If feasible, a larger sample size of donkeys will be employed to validate the findings of this study.

**Figure 9:**
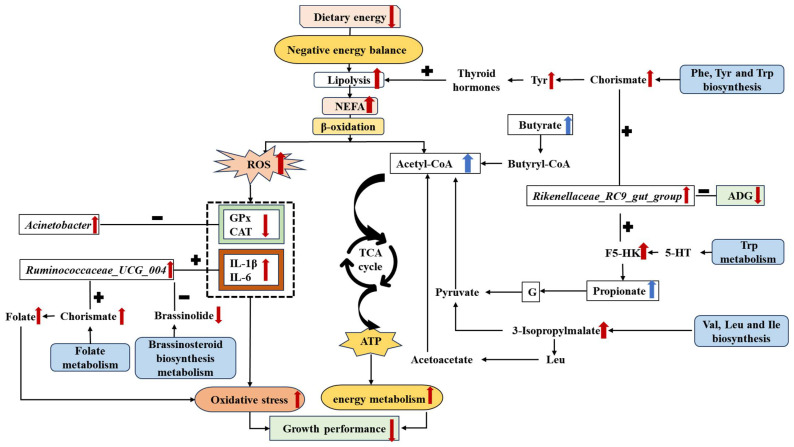
A mechanism map for linking the microbiota and the metabolites involved in the metabolic pathway of enrichment. Abbreviations: BW = body weight; NEFAs = non-esterified fatty acids; ROS = reactive oxygen species; G = glucose; CAT = catalase; GPx = glutathione peroxidase; IL-1β = interleukin-1β; IL-6 = interleukin-6; TNF-α = tumor necrosis factor-alpha; 5-HT = 5-hydroxytryptamine; f5-HK = formyl-5-hydroxykynurenine; Butyryl-CoA = butyryl coenzyme A; Acetyl-CoA = acetyl coenzyme A; Trp = tryptophan; Val = valine; Leu = leucine; Ile = isoleucine; Phe = phenylalanine; Tyr = tyrosine; TCA cycle = tricarboxylic acid cycle. “+” indicates a positive correlation between microorganisms and metabolites or other substances, and “−” indicates a negative correlation. The blue boxes represent enriched differential metabolic pathways in E2 (high dietary energy) vs. E1 (low dietary energy). “ 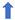 ” represents a positive increase, “ 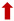 ” represents a negative increase, and “ 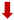 ” represents a negative decrease.

Based on the above analysis, Figure 9 provides a mechanism for linking the microbiota and the metabolites involved in the metabolic pathway of enrichment. In brief, lower dietary energy levels led to a negative energy balance in the donkeys, resulting in increased lipid metabolism and the production of more NEFAs. To compensate for the lack of dietary energy, NEFA was oxidized to generate ROS and acetyl-CoA. ROS induced oxidative stress in the donkeys, while acetyl-CoA entered the TCA cycle for energy production. As a result, more nutrients in the low-energy group were utilized for energy provision or the alleviation of oxidative stress, leading to reduced growth rates. This sequence of metabolic responses was modulated by cecal microbes and metabolites. Similarly, Li et al. [12] demonstrated that microbiome abundance and composition changes affect host metabolism. However, the causal relationship and mechanisms between the microbiome and metabolome still need further study and exploration.

## 5. Conclusions

Low-energy diets diminished the growth performance and nutrient digestion of donkeys, which may be related to low energy leading to a negative energy balance, oxidizing more nutrients for energy supply and further causing oxidative stress. And the reduction in the growth of meat donkeys due to low energy was associated with variations in cecal bacterial flora and metabolites. Low-energy diets altered microorganisms and metabolites in the cecum involved in pathways that induce oxidative stress and enhance energy metabolism. This study provided new perspectives for developing dietary strategies to improve the productivity of donkeys in feeding systems.

## Figures and Tables

**Table 1:** Diet composition and nutrition level (dry matter basis, %).

Item	PF ^1^		MF ^2^		LF ^3^	
	E1 ^4^	E2 ^5^	E1	E2	E1	E2
Ingredient						
Millet straw	55.18	43.34	40.28	35.49	38.34	33.70
Alfalfa	2.12	11.84	4.02	6.03	2.04	4.06
Corn silage	6.43	9.77	6.09	9.13	5.55	8.30
Corn	6.03	14.82	27.12	30.45	37.43	36.52
Wheat middling	0.00	0.00	1.60	2.00	1.76	1.92
Soybean meal	8.40	8.00	7.20	6.40	6.22	3.96
Corn gluten meal	0.60	0.92	0.70	0.00	0.00	0.00
Corn germ meal	7.42	1.40	3.00	0.00	0.00	0.00
DDGS	1.60	3.40	3.16	3.76	3.47	2.20
Bran	10.00	3.90	3.10	0.00	0.00	0.00
Soybean oil	0.00	0.40	0.00	0.00	0.00	0.82
Puffed full-fat soybeans	0.00	0.00	1.00	4.00	2.20	5.50
NaCl	0.40	0.40	0.50	0.50	0.55	0.55
Limestone	0.44	0.44	0.56	0.56	0.61	0.61
CaHPO_3_	0.88	0.88	1.10	1.10	1.21	1.21
Premix ^6^	0.20	0.20	0.20	0.20	0.20	0.20
NaHCO_3_	0.30	0.30	0.38	0.38	0.44	0.44
Total	100.00	100.00	100.00	100.00	100.00	100.00
Chemical composition, %						
Digestible energy, MJ/kg ^7^	12.08	13.38	13.01	14.27	13.54	14.93
Crude protein	14.53	14.64	13.02	13.04	12.48	12.67
Ether extract	5.69	6.43	6.06	6.50	6.47	6.95
Neutral detergent fiber	57.29	56.38	48.01	46.70	46.94	43.86
Acid detergent fiber	37.90	38.41	31.19	30.85	31.95	30.29
Calcium	1.33	1.38	1.48	1.45	1.36	1.40
Phosphorus	0.56	0.57	0.61	0.60	0.57	0.61

^1^ PF = pre-fattening. ^2^ MF = mid-fattening. ^3^ LF = late fattening. ^4^ E1 = low-energy group. ^5^ E2 = high-energy group. ^6^ Provided per kilogram of premix: Fe 20 g, Cu 5 g, Zn 20 g, Mn 20 g, I 120 mg, Se 120 mg, Co 120 mg, VitA 2,000,000 IU, VitD 3,800,000 IU, and VitE 7000 IU. ^7^ Digestible energy was calculated, and other chemical compositions were measured.

**Table 2:** Effects of dietary energy level on the growth performance of meat donkeys.

FST ^1^	Energy ^2^	BW, kg ^3^	DMI, kg/d ^4^	ADG, g/d ^5^	F-G ^6^
PF	E1	170.27	3.56	402.06	8.90
	E2	173.53	3.86	457.28	8.45
MF	E1	180.50	4.75	541.75	8.78
	E2	198.44	4.83	865.60	5.59
LF	E1	205.87	5.15	393.07	13.21
	E2	230.18	5.16	484.72	10.62
FST	PF	171.90	3.71	429.67	8.67
	MF	189.47	4.79	703.68	7.18
	LF	218.02	5.15	438.90	11.91
Energy	E1	185.55 ^B^	4.49	445.63 ^B^	10.30 ^A^
	E2	200.72 ^A^	4.62	602.53 ^A^	8.22 ^B^
SEM ^7^		4.076	0.097	4.389	0.273
*p*-value	Energy	0.017	0.354	<0.001	<0.001
	FST	<0.001	<0.001	<0.001	<0.001
	Energy × FST	0.159	0.672	<0.001	0.018

^1^ FST = fattening stage; PF = pre-fattening; MF = mid-fattening; LF = late fattening. ^2^ E1 = low-energy group; E2 = high-energy group. ^3^ BW=body weight. ^4^ DMI=dry matter intake. ^5^ ADG = average daily gain.^6^ F/G = the ratio of DMI to ADG. ^7^ SEM = standard error of least square means. ^AB^ In the same column, values with different capital letters indicate significant differences between the two groups (*p* < 0.05).

**Table 3:** Effects of dietary energy level on the nutrient digestibility and serum biochemical parameters of meat donkeys.

Item ^1^	E1 ^2^	E2 ^3^	SEM ^4^	*p*-Value
Nutrient digestibility, %				
CP	64.2 ^B^	68.3 ^A^	0.87	0.007
EE	57.1 ^B^	74.3 ^A^	1.95	<0.001
NDF	49.0 ^B^	58.2 ^A^	1.95	0.008
ADF	37.4 ^B^	47.6 ^A^	2.37	0.013
Serum biochemical parameters, mmol/L				
GLU	4.64	5.14	0.13	0.139
CHO	1.74 ^B^	2.01 ^A^	0.08	0.039
UREA	2.25	2.02	0.09	0.095
NEFA	0.33 ^A^	0.26 ^B^	0.00	<0.001

^1^ CP = crude protein; EE = ether extract; NDF = neutral detergent fiber; ADF = acid detergent fiber; GLU = glucose; CHO = cholesterol; UREA = urea nitrogen and NEFAs = non-esterified fatty acids. ^2^ E1 = low-energy group. ^3^ E2 = high-energy group. ^4^ SEM = standard error of least square means. ^AB^ Means within the same row followed by the same superscript letters are not significantly different at *p* < 0.05.

**Table 4:** Effects of dietary energy level on VFAs in meat donkeys.

VFA ^1^	E1 ^2^	E2 ^3^	SEM ^4^	*p* Value
Total, mmol/L	49.41 ^A^	37.31 ^B^	1.979	0.001
Acetate, mmol/L	36.53 ^A^	26.44 ^B^	0.523	0.001
Propionate, mmol/L	8.92 ^A^	7.51 ^B^	0.393	0.016
A/P ratio	4.12 ^A^	3.51 ^B^	0.394	0.020
Butyrate, mmol/L	2.74 ^A^	2.39 ^B^	0.154	0.003
Isobutyrate, mmol/L	0.39 ^A^	0.30 ^B^	0.035	0.001
Isovalerate, mmol/L	0.44 ^A^	0.36 ^B^	0.031	0.044
Valerate, mmol/L	0.39 ^A^	0.31 ^B^	0.055	0.015

^1^ VFA = volatile fatty acid; A/P = the ratio of acetate to propionate. ^2^ E1 = low-energy group. ^3^ E2 = high-energy group. ^4^ SEM = standard error of least square means. ^AB^ Means within the same row followed by the same superscript letters are not significantly different at *p* < 0.05.

**Table 5:** Effect of dietary energy levels on digestive tract mucosal enzyme activity in meat donkeys.

Item	E1 ^1^	E2 ^2^	SEM ^3^	*p*-Value
α-amylase activity, ng/m				
Duodenum	7.45	8.55	0.342	0.062
Jejunum	10.05 ^B^	12.21 ^A^	0.417	0.006
Ileum	9.38 ^B^	11.01 ^A^	0.340	0.010
Chymotrypsin activity, pg/mL				
Duodenum	400.04	407.46	5.400	0.344
Jejunum	389.25	380.86	6.809	0.395
Ileum	390.86	395.35	6.905	0.651
Trypsin activity, ng/mL				
Duodenum	202.00 ^B^	231.00 ^A^	8.466	0.026
Jejunum	187.07 ^B^	212.67 ^A^	2.515	<0.001
Ileum	196.78 ^B^	219.39 ^A^	5.345	0.008
Lipase activity, ng/mL				
Duodenum	81.80 ^B^	89.58 ^A^	2.594	<0.001
Jejunum	47.24 ^B^	64.23 ^A^	1.75	<0.001
Ileum	41.81 ^B^	54.18 ^A^	1.709	<0.001

^1^ E1 = low-energy group. ^2^ E2 = high-energy group. ^3^ SEM = standard error of least square means. ^AB^ Means within the same row followed by the same superscript letters are not significantly different at *p* < 0.05.

**Table 6:** Effects of dietary energy level on serum antioxidant activities and immune signaling molecule levels of meat donkeys.

Item	E1 ^1^	E2 ^2^	SEM ^3^	*p*-Value
Antioxidant enzyme activities, U/mL				
CAT	8.26 ^B^	11.89 ^A^	0.199	<0.001
GPx	433.33 ^B^	500.67 ^A^	6.673	<0.001
T-SOD	136.76 ^A^	122.51 ^B^	2.356	0.001
Immune signaling molecule, pg/mL				
IL-1β	26.17 ^A^	21.14 ^B^	0.783	0.001
IL-2	268.73 ^A^	233.55 ^B^	4.244	<0.001
IL-6	155.97 ^A^	132.95 ^B^	1.826	<0.001
IL-4	6.53 ^B^	7.80 ^A^	0.159	<0.001
IL-10	8.30 ^B^	12.13 ^A^	0.204	<0.001
TNF-α	70.48 ^A^	46.67 ^B^	1.978	<0.001
MDA concentration, nmol/mL	2.16 ^A^	1.92 ^B^	0.041	0.001
NO concentration, umol/L	40.20	39.65	0.573	0.503
ROS concentration, IU/mL	131.00 ^A^	119.32 ^B^	1.218	<0.001

^1^ E1 = low-energy group. ^2^ E2 = high-energy group. ^3^ SEM = standard error of least square means. ^AB^ Means within the same row followed by the same superscript letters are not significantly different at *p* < 0.05. Abbreviations: CAT = catalase; GPx = glutathione peroxidase; T-SOD = total superoxide dismutase; MDA = malondialdehyde; IL = interleukin; TNF-α = tumor necrosis factor-alpha; NO = nitric oxide; and ROS = reactive oxygen species.

**Table 7:** Effects of dietary energy level on cecal antioxidant activities and immune signaling molecule levels of meat donkeys.

Item	E1 ^1^	E2 ^2^	SEM ^3^	*p*-Value
Antioxidant enzyme activities, U/mgprot				
CAT	37.10 ^B^	40.29 ^A^	0.835	0.017
GPx	6.50	6.84	0.138	0.109
T-SOD	74.23 ^B^	94.49 ^A^	1.670	<0.001
Immune signaling molecule, pg/mgprot				
IL-1β	3.02 ^A^	2.67 ^B^	0.083	0.010
IL-2	48.53 ^A^	39.56 ^B^	0.654	<0.001
IL-6	26.94 ^A^	23.45 ^B^	0.532	<0.001
IL-	0.73 ^B^	0.81 ^A^	0.015	0.002
IL-1	1.40 ^B^	1.59 ^A^	0.021	<0.001
TNF-α	5.00 ^A^	4.21 ^B^	0.161	0.004
MDA concentration, nmol/mgprot	1.78	1.56	0.133	0.259
NO concentration, umol/gprot	6.77 ^B^	7.68 ^A^	0.069	<0.001
ROS concentration, IU/mgprot	342.88 ^A^	276.98 ^B^	8.475	<0.001

^1^ E1 = low-energy group. ^2^ E2 = high-energy group. ^3^ SEM = standard error of least square means. ^AB^ Means within the same row followed by the same superscript letters are not significantly different at *p* < 0.05. Abbreviations: CAT = catalase; GPx = glutathione peroxidase; T-SOD = total superoxide dismutase; MDA = malondialdehyde; IL = interleukin; TNF-α = tumor necrosis factor-alpha; NO = nitric oxide; and ROS = reactive oxygen species.

**Table 8:** Effects of dietary energy level on microflora composition at the phylum level, %.

Item	E1 ^1^	E2 ^2^	SEM ^3^	*p* Value
*Firmicutes*	60.62 ^A^	51.80 ^B^	2.440	0.029
*Bacteroidetes*	30.28 ^B^	39.59 ^A^	1.841	0.005
*Actinobacteria*	0.68 ^A^	0.21 ^B^	0.082	0.002
*Spirochaetes*	3.40	3.66	1.069	0.868
*Tenericutes*	0.40	0.33	0.048	0.304
*Proteobacteria*	0.55	0.64	0.089	0.493

^1^ E1 = low-energy group. ^2^ E2 = high-energy group. ^3^ SEM = standard error of least square means. ^AB^ Means within the same row followed by the same superscript letters are not significantly different at *p* < 0.05.

**Table 9:** Particular significant metabolites for E2 vs. E1.

Metabolites	HMDB Superclass	VIP ^1^	FC ^2^	*p*-Value
PGA3	Others	1.949	0.974	0.020
L-Norvaline	Others	1.870	0.960	0.017
Brassinolide	Lipids and lipid-like molecules	1.896	1.034	0.017
Cinnamaldehyde	Phenylpropanoids and polyketides	1.947	0.967	0.014
Sucrose	Organic oxygen compounds	2.291	0.959	0.005
Kanzonol N	Phenylpropanoids and polyketides	2.105	0.979	0.006
Chorismate	Organic acids and derivatives	1.919	0.935	0.016
3-Isopropylmalate	Lipids and lipid-like molecules	1.746	0.958	0.040
3-Sulfinoalanine	Organic acids and derivatives	1.798	1.047	0.029
Formyl-5-hydroxykynurenamine	Organic oxygen compounds	1.853	0.965	0.020
Indole-3-acetic-acid-O-glucuronide	Organic oxygen compounds	2.045	0.951	0.008
7-Methoxy-2-methylisoflavone	Phenylpropanoids and polyketides	1.786	0.906	0.029
Urolithin C	Phenylpropanoids and polyketides	1.714	0.874	0.038
N-lactoyl-Phenylalanine	Organic acids and derivatives	1.858	0.894	0.020
4-Hydroxybenzoic acid	Benzenoids	1.849	0.910	0.022
3-(2-(methylamino)ethyl)-1H-indol-5-ol	Others	1.859	0.858	0.022

^1^ VIP = VIP value from the OPLS-DA model; ^2^ FC = fold change ratio E2 vs. E1; FC > 1 indicates that the substance is significantly elevated in the E2; and FC < 1 indicates the opposite. E1 = low-energy group; E2 = high-energy group.

**Table 10:** Enrichment pathways of differential metabolites for E2 vs. E1.

Metabolic Pathways	Hits ^1^	*p*-Value ^2^	Upregulated	Downregulated
Tryptophan metabolism	2	0.013		Formyl-5-hydroxykynurenamine 3-(2-(methylamino)ethyl)- 1H-indol-5-ol
Folate biosynthesis	2	0.014		4-Hydroxybenzoic acidChorismate
Taurine and hypotaurine metabolism	1	0.069	3-Sulfinoalanine	
Valine, leucine, and isoleucine biosynthesis	1	0.078		3-Isopropylmalate
Phenylalanine, tyrosine, and tryptophan biosynthesis	1	0.111		Chorismate
Starch and sucrose metabolism	1	0.114		Sucrose
Galactose metabolism	1	0.144		Sucrose
Ubiquinone and other terpenoid–quinone biosynthesis	2	0.013		4-Hydroxybenzoic acid Chorismate
Brassinosteroid biosynthesis	1	0.087	Brassinolide	

^1^ Hits represents the number of KEGG compound IDs annotated to pathways in this metabolism process. ^2^ The *p*-value is uncorrected, and *p*-values less than 0.05 are considered enrichment terms.

## Data Availability

The datasets presented in this study can be found in online repositories. The names of the repository/repositories and accession number(s) can be found at NCBI SRA; PRJNA1119722.

## Supplementary Materials

The following supporting information can be downloaded at: https://www.mdpi.com/article/10.3390/antiox13111377/s1, Figure S1A: OTU rarefaction curves of cecal digesta bacterial communities; Figure S1B: PCoA (using the Bray–Curtis similarity metric) of bacterial OTUs in the cecal contents of meat donkeys; Figure S2A: PCA score plots of E2 vs. E1 in Pos. Figure S2B: PCA score plots of E2 vs. E1 in Neg. Figure S2C: OPLS-DA score plots of E2 vs. E1 in Pos. Figure S2D: OPLS-DA score plots of E2 vs. E1 in Neg. Figure S2E: permutation test of OPLS-DA model for group E2 vs. E1 in Pos. Figure S2F: permutation test of OPLS-DA model for group E2 vs. E1 in Neg. Table S1: effects of dietary energy level on cecal microbe alpha diversity in meat donkeys.
