# Supplementary material for: Cecal Microbial Diversity and Metabolome Reveal a Reduction in Growth Due to Oxidative Stress Caused by a Low-Energy Diet in Donkeys
Source: Antioxidants (Basel). 2024 Nov 11;13(11):1377. doi: 10.3390/antiox13111377 (PMC11591121; doi:10.3390/antiox13111377)
Supplement: Supplementary file 1 [file antioxidants-13-01377-s001.zip › Supplementary-tableS1.pdf]

Cecal microbial diversity and metabolome reveal a reduction in growth due to oxidative stress caused by a low-energy diet in donkeys

Li L [1], Xiaoyu Guo [1], Yanli Zhao [1], Yongmei Guo [1], Binlin Shi [1], Yan Zhou [1], Yongwei Zhang [2] and Sumei Yan [1,*]

[1] Inner Mongolia Key Laboratory of Animal Nutrition and Feed Science, College of Animal Science, Inner Mongolia Agricultural University, Hohhot 010018, China; lily972021@163.com (L.L.); gxy_2594@163.com (X.G.); ylzhao2010@163.com (Y.Z.); ymguo2015@163.com (Y.G.); shibinlin@yeah.net (B.S.); 1454803209@qq.com (Y.Z.)

[2] Inner Mongolia Grassland Yulv Science and Technology Animal Husbandry Co., Ltd. Horinger County 011500, China. 1010142628@qq.com (Y.Z.)

* Correspondence: yansmimau@163.com

Table S1. Effects of dietary energy level on cecal microbe alpha diversity of meat donkeys

**Alpha diversity index**	**E1[1]**	**E2[2]**	**SEM[3]**	***P value***
Sobs	1071.00	1024.25	37.645	0.401
Chao	1413.81	1249.22	58.989	0.077
Ace	1365.02	1246.08	55.716	0.162
Shannon	5.46	5.44	0.064	0.815
Simpson	0.01	0.01	0.002	0.950
Coverage	0.99	0.99		

[1]E1=low-energy group.

[2]E2=high-energy group.

[3]SEM: standard error of least square means.
