# Supplementary material for: Cecal Microbial Diversity and Metabolome Reveal a Reduction in Growth Due to Oxidative Stress Caused by a Low-Energy Diet in Donkeys
Source: Antioxidants (Basel). 2024 Nov 11;13(11):1377. doi: 10.3390/antiox13111377 (PMC11591121; doi:10.3390/antiox13111377)
Supplement: Supplementary file 1 [file antioxidants-13-01377-s001.zip › Supplementary-figureS1.pdf]

Cecal microbial diversity and metabolome reveal a reduction in growth due to oxidative stress caused by a low-energy diet in donkeys

Li L [1], Xiaoyu Guo [1], Yanli Zhao [1], Yongmei Guo [1], Binlin Shi [1], Yan Zhou [1], Yongwei Zhang [2] and Sumei Yan [1,*]

[1] Inner Mongolia Key Laboratory of Animal Nutrition and Feed Science, College of Animal Science, Inner Mongolia Agricultural University, Hohhot 010018, China; lily972021@163.com (L.L.); gxy_2594@163.com (X.G.); ylzhao2010@163.com (Y.Z.); ymguo2015@163.com (Y.G.); shibinlin@yeah.net (B.S.); 1454803209@qq.com (Y.Z.)

[2] Inner Mongolia Grassland Yulv Science and Technology Animal Husbandry Co., Ltd. Horinger County 011500, China. 1010142628@qq.com (Y.Z.)

* Correspondence: Correspondence: yansmimau@163.com

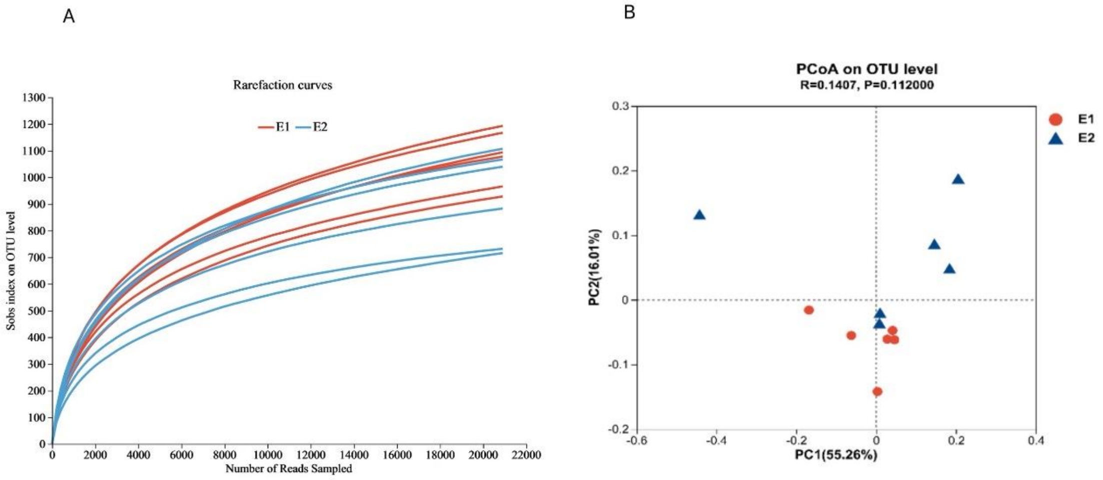

Figure S1. (A) The OTU rarefaction curves of the cecal digesta bacterial communities. Curves are drawn using the least sequenced sample as the upper limit for the rarefactions. (B) PCoA (using the Bray–Curtis similarity metric) of bacterial OTU in the cecal contents of meat donkeys. Each color represents one treatment: red curves represent donkeys fed a low-energy (E1) diet and the blue curves represent donkeys fed with a high-energy (E2) diet.
